# Detection of porcine parainfluenza virus type-1 antibody in swine serum using whole-virus ELISA, indirect fluorescence antibody and virus neutralizing assays

**DOI:** 10.1186/s12917-022-03196-6

**Published:** 2022-03-21

**Authors:** Michael Welch, Karen Krueger, Jianqiang Zhang, Pablo Piñeyro, Ronaldo Magtoto, Chong Wang, Luis Giménez-Lirola, Erin Strait, Mark Mogler, Phillip Gauger

**Affiliations:** 1 Department of Veterinary Diagnostic and Production Animal Medicine, College of Veterinary Medicine, Iowa State University, 1800 Christensen Drive, Ames, IA 50011 USA; 2 Department of Statistics, College of Liberal Arts and Sciences, Iowa State University, 2438 Osborn Drive, Ames, IA 50011 USA; 3 Merck Animal Health, Ames, IA USA; 4 Ceva Animal Health, LLC, 8901 Rosehill Road, Lenexa, KS 66215 USA

**Keywords:** Porcine parainfluenza virus 1, Validation, Serology, Serum virus neutralization, Indirect fluorescence antibody, Enzyme linked immunosorbent assay

## Abstract

**Background:**

Porcine parainfluenza virus 1 (PPIV-1) is a respiratory virus in the family *Paramyxoviridae* and genus *Respirovirus*. It is closely related to bovine parainfluenza virus 3, human parainfluenza virus 1, and Sendai virus. Recent reports suggest PPIV-1 is widespread in swine herds in the United States and abroad. However, seroprevalence studies and the ability to evaluate cross neutralization between heterologous strains is not possible without validated antibody assays. This study describes the development of an indirect fluorescence antibody (IFA) assay, a whole virus enzyme-linked immunosorbent assay (wv-ELISA) and a serum virus neutralization (SVN) assay for the detection of PPIV-1 antibodies using 521 serum samples collected from three longitudinal studies and two different challenge strains in swine.

**Results:**

The area under the curve (AUC) of the wv-ELISA (95% CI, 0.93–0.98) was significantly higher (*p* = 0.03) compared to the IFA (95% CI, 0.90–0.96). However, no significant difference was observed between the IFA and wv-ELISA when compared to the SVN (95% CI, 0.92–0.97). All three assays demonstrated relatively uniform results at a 99% true negative rate, with only 11 disagreements observed between the IFA, wv-ELISA and SVN.

**Conclusions:**

All three serology assays detected PPIV-1 antibody in swine serum of known status that was collected from experimental studies. The SVN detected seroconversion earlier compared to the IFA and the wv-ELISA. Both the wv-ELISA and the SVN had similar diagnostic performance, while the IFA was not as sensitive as the wv-ELISA. All three assays are considered valid for routine diagnostic use. These assays will be important for future studies to screen seronegative swine for research, determine PPIV-1 seroprevalence, and to evaluate vaccine efficacy against PPIV-1 under experimental and field conditions.

## Background

Porcine parainfluenza virus-1 (PPIV-1) is a newly characterized respiratory virus in the family *Paramyxoviridae*, genus *Respirovirus*. Previously identified paramyxoviruses that cause clinical disease in swine include blue eye paramyxovirus (La Piedad-Michoacan virus), Menangle virus, and Nipah virus [1]. However, none of these three paramyxoviruses have been detected in United States (U.S.) commercial swine [1]. PPIV-1 was originally discovered from slaughter swine in Hong Kong by L-gene specific reverse transcription real time polymerase chain reaction (RT-rtPCR) and complete genome sequencing [2]. Due to the difficulty of isolating PPIV-1 in cell culture, initial studies investigating the pathogenesis of PPIV-1 were limited to pigs obtained from naturally infected herds [3]. Retrospective studies evaluating respiratory samples from clinically affected pigs by RT-rtPCR showed a 43.3% cumulative positive rate in a variety of sample types including oral fluids (67.6%), nasal swabs (64.1%), nasal turbinate (50%), and bronchioalveolar lavage (50%) [4]. These data suggest that PPIV-1 is widespread in U.S. swine.

A PPIV-1 isolate was obtained using a MK-2 cell line (ATCC CCL-7™) derived from rhesus monkey kidney epithelium [4]. The isolate was inoculated intranasally and intratracheally at approximately 10^5^ 50% tissue culture infectious dose per milliliter (TCID_50_/mL) into three-week-old (nursery-age), conventional and six-week-old cesarean derived-colostrum deprived (CDCD) piglets [5]. Virus was detected in high quantities (> 10^5^ genomic copies/mL) in all respiratory samples by RT-rtPCR, and in particular from tracheal mucosal swabs. Mild respiratory clinical signs were observed in spite of high levels of viral replication evidenced by detection of viral nucleic acid in nasal swabs, bronchoalveolar lavage fluid, tracheal mucosal swabs, and successful virus isolation. In addition to the U.S. and Hong Kong, PPIV-1 has been reported in European and South American swine, specifically in Chile and Hungary [6, 7] with a 9.09% and 18.90% farm-level prevalence, respectively. Phylogenetic analysis determined that the Chilean sequences clustered more closely with those detected from China in contrast to North American sequences, forming a separate monophyletic clade [7]. Similarly, PPIV-1 sequences from swine in Hungary were more genetically related to Chinese PPIV-1 even though the frequency of detection from these herds was much lower [6]. However, differences in detection rate could be attributed to inter-laboratory variation, sampling methodologies and differences in test sensitivity.

Information regarding the PPIV-1 seroprevalence in the field or the humoral antibody response under natural or experimental conditions is lacking. Thus far, an indirect ELISA using a recombinant fusion (F) protein peptide is the only assay that has been described to evaluate seroconversion, but was only validated using samples from naturally infected pigs [3]. The ELISA detected anti-F IgG antibodies in seven out of eleven pigs on day 0 (22–23 days of age) from this field study. It was hypothesized that the PPIV-1 antibody positive results were due to waning, passively-acquired maternal antibodies, but the true status of the samples could not be confirmed. These results demonstrate the importance of using samples of defined status in order to rigorously validate anti-PPIV-1 antibody assays. However, due to the widespread nature of PPIV-1, it is difficult to obtain samples of known status for assay validation. This study describes the validation of three, anti-PPIV-1 antibody assays using sera of known status that was determined by comparing antibody responses from experimentally challenged or vaccinated pigs to known negative controls. Validated serology assays are needed for estimating PPIV-1 seroprevalence, screening for the presence of maternal antibodies, and evaluating vaccine efficacy.

## Results

### Comparison of wv-ELISA, IFA, and SVN PPIV-1 antibody assays

**Fig. 1:**
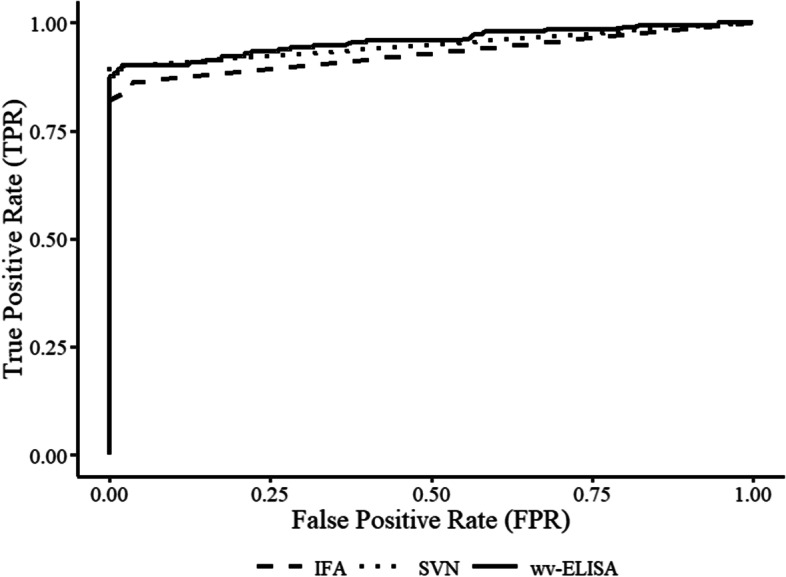
Comparative receiver operating characteristic (ROC) analysis. Area under the curve (AUC) was compared between assays. The graph demonstrates the wv-ELISA AUC was significantly increased relative to the IFA (0.96 vs 0.93). However, no significant difference was observed between the SVN and wv-ELISA (0.95 vs 0.96) or SVN and IFA (0.93 vs 0.95)

An empirical receiver operating characteristic (ROC) analysis was performed on a subset of 372 serum (196 exposed and 176 non-exposed) obtained from experimentally inoculated or RNA Particle (RP)-vaccinated pigs for estimation of optimal cut-off and associated diagnostic sensitivity and specificity. A subset of the 521 serum was chosen to allow time for adequate seroconversion. Regarding the sera of known status, 24 originated from Study A (16 pos, 8 neg), 48 from Study B (30 pos, 18 neg), and 300 from Study C (150 pos, 150 neg). True positive rates (TPR) and true negative rates (TNR) were reported over a range of thresholds to help determine an appropriate assay cut-off (Table 1). Area under the curve (AUC) was calculated and compared using the pROC library in R (Fig. 1) [8]**.** The wv-ELISA AUC (95% CI, 0.93–0.98) was significantly higher (*p* = 0.0318) compared to the IFA (95% CI 0.90–0.96). However, no significant difference was observed between either the IFA or wv-ELISA compared to the SVN (95% CI, 0.92–0.97).

**Table 1:** Selected thresholds, true positive rates (TPR) and true negative rates (TNR) for the Porcine Parainfluenza Virus 1 wv-ELISA, IFA and SVN assays. Bootstrapped 95% confidence intervals are provided in parentheses when estimable

Assay	Threshold	TPR %	TNR %
wv-ELISA	0.03	93 (89–97)	75 (69–83)
	0.10	90 (86–94)	97 (94–99)
	0.14	88 (83–92)	99 (97–100)
IFA	20	87 (81–92)	94 (87–99)
	40	86 (80–92)	97 (91–100)
	80	82 (75–88)	> 99
	160	69 (56–80)	> 99
SVN	10	90 (85–94)	> 99
	20	79 (70–87)	> 99
	40	69 (56–80)	> 99

**Fig. 2:**
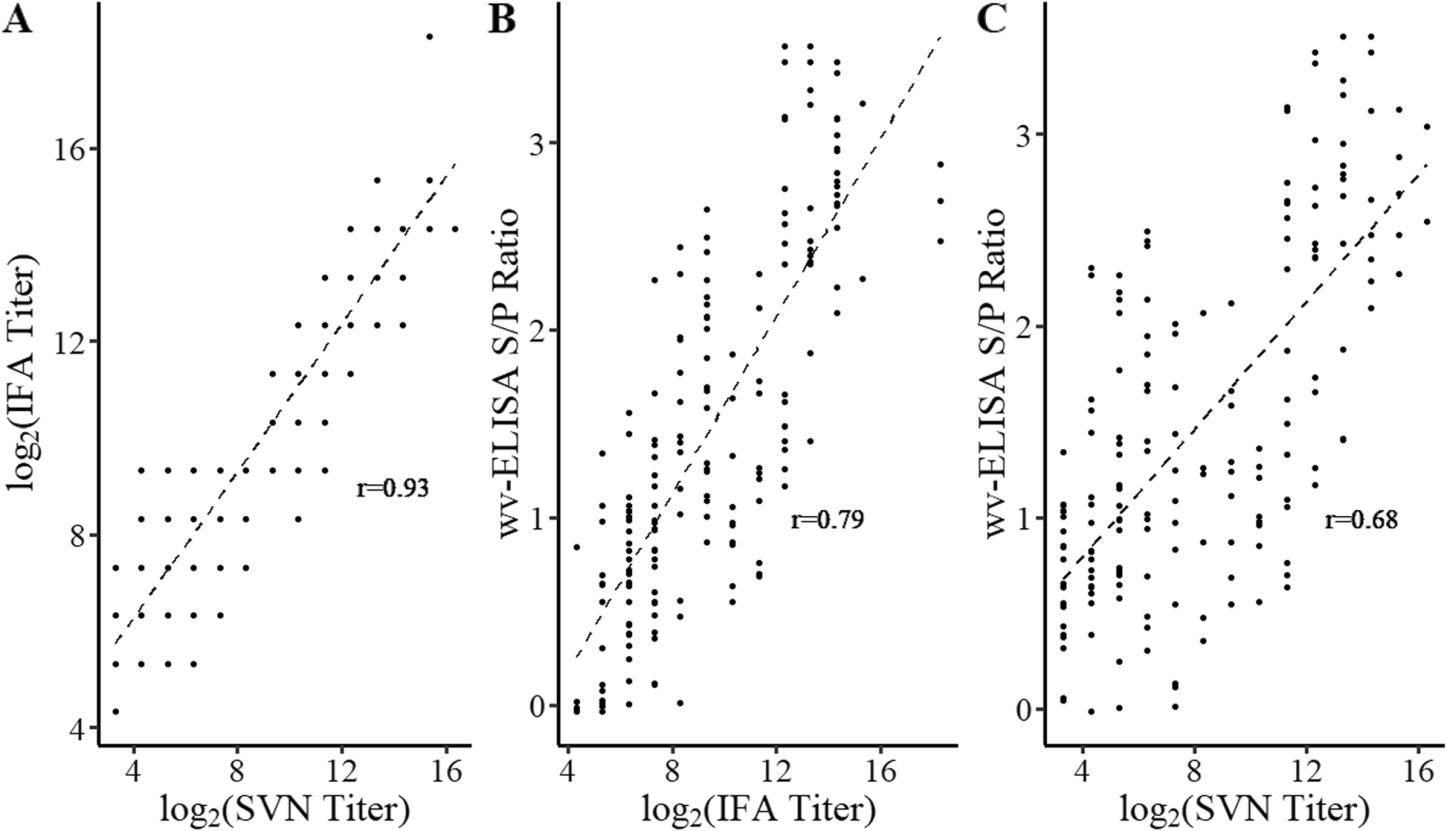
Correlation of antibody concentration among positive samples and percent agreement at a 99% true negative rate. **A** IFA titer and SVN titer, **B** IFA titer and wv-ELISA S/P ratio and **C** wv-ELISA S/P ratio and SVN titer. Figure 2A demonstrated the highest correlation (*r* = 0.93) between IFA and SVN assays. Similarly, Fig. 2B demonstrated a strong correlation (*r* = 0.79). The correlation between SVN and wv-ELISA in 2C was lowest at 0.69. SVN: serum virus neutralization, IFA: indirect fluorescence assay, wv-ELISA: whole virus enzyme linked immunosorbent assay

The correlation between SVN and IFA titer was highest at *r* = 0.93 (Fig. 2A). A strong correlation was observed between the IFA and wv-ELISA (0.79) (Fig. 2B) and a moderate correlation was observed between the SVN and wv-ELISA (0.68) results (Fig. 2C). All three assays demonstrated relatively uniform results at 99% TNR where 175 sera were determined negative by the three assays and 197 sera determined positive for PPIV-1 antibody. For the 372 samples selected for the ROC analysis, only 3.0% disagreement was observed between the SVN and IFA (Table 2) as well as between the IFA and wv-ELISA (Table 3). The SVN and wv-ELISA disagreement was lower at 2.2% (Table 4).

**Table 2:** Correlation between IFA and SVN PPIV1 antibody titers. A high agreement was observed between the two assays with 11 positive IFA and SVN samples not detected by the alternate test. The top and bottom numbers in each well represent the row, column, and total percentages, respectively

		IFA			
SVN	Neg		Pos	Total	
Neg	186		11	197	
	94.42%		5.58%	100%	
	94.42%		6.29%	52.96%	
	50.00%		2.96%	100%	
Pos	11		164	175	
	6.29%		93.71%	100%	
	5.58%		93.71%	47.04%	
	2.96%		44.09%	100%	
Total	197		175	372	
	52.96%		47.04%	100 (row%)	
	100%		100%	100 (col%)	
	100%		100%	100 (tot%)	

*SVN* Serum virus neutralization, *IFA* Indirect fluorescence antibody assay

**Table 3:** Correlation between IFA titer and wv-ELISA S/P ratio. A high agreement was observed between the two assays, with 11 positive IFA and wv-ELISA samples not detected by the alternate test. The top and bottom numbers in each well represent the row, column, and total percentages, respectively

	IFA		
wv-ELISA	Neg	Pos	Total
Neg	186	11	197
	94.42%	5.58%	100%
	94.42%	6.29%	52.96%
	50.00%	2.96%	100%
Pos	11	164	175
	6.29%	93.71%	100
	5.58%	93.71%	47.04%
	2.96%	44.09%	100%
Total	197	175	372
	52.96%	47.04%	100 (row%)
	100%	100%	100 (col%)
	100%	100%	100 (tot%)

*IFA* Indirect fluorescence antibody assay, *wv-ELISA* Whole virus enzyme linked immunosorbent assay

**Table 4:** Correlation between SVN titer and wv-ELISA S/P ratio. The highest agreement was observed between the two assays, with only 8 positive IFA and wv-ELISA samples not detected by the alternate test. The top and bottom numbers in each well represent the row, column, and total percentages, respectively

	wv-ELISA		
SVN	Neg	Pos	Total
Neg	189	8	197
	95.94%	4.06%	100
	95.94%	4.57%	52.96%
	50.81%	2.15%	100%
Pos	8	167	175
	4.57%	95.43%	100
	4.06%	95.43%	47.04%
	2.15%	44.89%	100%
Total	197	175	372
	52.96%	47.04%	100 (row%)
	100%	100%	100 (col%)
	100%	100%	100 (tot%)

*SVN* Serum virus neutralization, *wv-ELISA* Whole virus enzyme linked immunosorbent assay

#### Comparison of assay threshold by treatment and timepoint

Results from three separate experimental studies are reported across experimental groups. Five-hundred twenty-one samples collected longitudinally from 75 piglets were included in the analysis. Assays within timepoint and group were compared using a Cochran’s Q test and individual pairwise McNemar test if significant. The highest proportion of seropositive samples was detected by SVN compared to wv-ELISA or IFA. Positive results were observed by SVN in pigs as early as 7 days post inoculation (DPI) in Studies A and B (Tables 5 and 6) and 11 days post vaccination (DPV) in the LE/C, RP/C and RPAdj/C groups observed in Study C (Table 7). Significance testing of assay type by group in studies A and B were not significant (Tables 5 and 6). In Study C, a significant association with non-significant pairwise McNemar tests were found on 18, 24, 38, 48 DPV in the LE/C group; 11 and 24 DPV in the RP/C group; and 11, 18 and 24 DPV in the RPAdj/C group (Table 7).

**Table 5:** Study A: Cesarean-derived colostrum-deprived (CDCD) piglet challenge study. Anti-PPIV-1 antibody positive pigs at inoculation and 7, 9, 13, and 21 days later. A total of 14 piglets were included in this study, and pigs were necropsied at 2 DPI (3 Ch, 1 NCh), 5 DPI (4 Ch, 1 NCh) and 27 DPI (3 Ch, 2 NCh). Treatment groups included a negative control and PPIV-1 USA/MN/25890NS/2016 (MN16) challenged groups. Assay thresholds were determined by ROC analysis at a specificity of 99% while maximizing sensitivity

		Days Post Inoculation (DPI)				
Treatment	Assay	0	7	9	13	21
Challenge	wv-ELISA^A^	0 (0/10)^1^	0 (0/3)	67 (2/3)	100 (3/3)	100 (3/3)
	IFA^B^	0 (0/10)	0 (0/3)	0 (0/3)	100 (3/3)	100 (3/3)
	SVN^C^	0 (0/10)	100 (3/3)	100 (3/3)	100 (3/3)	100 (3/3)
Control	wv-ELISA^A^	0 (0/4)	0 (0/2)	0 (0/2)	50 (1/2)	0 (0/2)
	IFA^B^	0 (0/4)	0 (0/2)	0 (0/2)	0 (0/2)	0 (0/2)
	SVN^C^	0 (0/4)	0 (0/2)	0 (0/2)	0 (0/2)	0 (0/2)

^A^Samples with a sample/positive (S/P) ratio greater than 0.14 were considered positive. Samples with a S/P ratio less than 0.14 were considered negative

^B^Samples with an IFA reciprocal titer greater than or equal to 40 were considered positive. Samples with an IFA titer less than 40 were considered negative

^C^Samples with a VN titer greater than or equal to 20 were considered positive. Samples with a VN titer less than 20 were considered negative

^1^Antibody positive/treatment group size; *wv-ELISA* Whole virus ELISA, *IFA* Indirect fluorescence antibody, *SVN* Serum virus neutralization

**Table 6:** Study B: Human parainfluenza type-1 and PPIV-1 isolate comparison study. Percent of anti-PPIV-1 antibody positive pigs at inoculation and 7, 10, 14, 17, 21, 24, and 28 days later. A total of 30 piglets were included in this study, and 19 were necropsied at 5 DPI (15 Ch, 4 NCh) and 11 necropsied at 28 DPI. Treatment groups included a negative control, human parainfluenza virus type-1 (HPIV-1) challenged, PPIV-1 USA/IA/84915LG/2017 (IA17), and PPIV-1 USA/MN/25890NS/2016 (MN16) challenged groups. Assay thresholds were determined by ROC analysis at a specificity of 99% while maximizing sensitivity

		Days Post Inoculation (DPI)							
Treatment	Assay	0	7	10	14	17	21	24	28
Control	wv-ELISA^A^	0 (0/2)^1^	0 (0/2)	0 (0/2)	0 (0/2)	0 (0/2)	0 (0/2)	0 (0/2)	0 (0/2)
	IFA^B^	0 (0/2)	0 (0/2)	0 (0/2)	0 (0/2)	0 (0/2)	0 (0/2)	0 (0/2)	0 (0/2)
	SVN^C^	0 (0/2)	0 (0/2)	0 (0/2)	0 (0/2)	0 (0/2)	0 (0/2)	0 (0/2)	0 (0/2)
HPIV-1	wv-ELISA^A^	0 (0/3)	0 (0/3)	0 (0/3)	0 (0/3)	0 (0/3)	0 (0/3)	0 (0/3)	0 (0/3)
	IFA^B^	0 (0/3)	0 (0/3)	0 (0/3)	0 (0/3)	0 (0/3)	0 (0/3)	0 (0/3)	0 (0/3)
	SVN^C^	0 (0/3)	0 (0/3)	0 (0/3)	0 (0/3)	0 (0/3)	0 (0/3)	0 (0/3)	0 (0/3)
IA17	wv-ELISA^A^	0 (0/3)	0 (0/3)	100 (3/3)	100 (3/3)	100 (3/3)	100 (3/3)	100 (3/3)	100 (3/3)
	IFA^B^	0 (0/3)	0 (0/3)	100 (3/3)	100 (3/3)	100 (3/3)	100 (3/3)	100 (3/3)	100 (3/3)
	SVN^C^	0 (0/3)	100 (3/3)	100 (3/3)	100 (3/3)	100 (3/3)	100 (3/3)	100 (3/3)	100 (3/3)
MN16	wv-ELISA^A^	0 (0/3)	0 (0/3)	33 (1/3)	67 (2/3)	100 (3/3)	100 (3/3)	100 (3/3)	100 (3/3)
	IFA^B^	0 (0/3)	0 (0/3)	100 (3/3)	100 (3/3)	100 (3/3)	100 (3/3)	100 (3/3)	100 (3/3)
	SVN^C^	0 (0/3)	100 (3/3)	100 (3/3)	100 (3/3)	100 (3/3)	100 (3/3)	100 (3/3)	100 (3/3)

^A^Samples with a sample/positive (S/P) ratio greater than 0.14 were considered positive. Samples with a S/P ratio less than 0.14 were considered negative

^B^Samples with an IFA reciprocal titer greater than or equal to 40 were considered positive. Samples with an IFA titer less than 40 were considered negative

^C^Samples with a VN titer greater than or equal to 20 were considered positive. Samples with a VN titer less than 20 were considered negative

^1^Antibody positive/treatment group size; *wv-ELISA* Whole virus ELISA, *IFA* Indirect fluorescence antibody, *SVN* Serum virus neutralization

**Table 7:** Study C: PPIV-1 vaccine study. Percent of anti-PPIV-1 antibody positive pigs at vaccination or exposure (0 DPV) and 11, 18, 24, 31, 38, 43, and 48 DPV. A total of 50 piglets were included in this study. Treatment groups included live exposure/challenged (LE/C), non-vaccinated/challenged (NV/C), non-vaccinated/non-challenged (NV/NC), RNA particle vaccinated/challenged (RP/C), and adjuvanted RNA particle/challenged (RPAdj/C). Assay thresholds were determined by ROC analysis at a specificity of 99% while maximizing sensitivity. Values within an experimental group by DPV with different superscripts were significantly different at *p* ≤ 0.05 by McNemar’s test. Asterisks indicate significant Cochran’s Q test by DPV but nonsignificant individual McNemar’s pairwise testing

		Days Post Vaccination (DPV)							
Treatment	Assay	0	11	18	24	31	38	43	48
LE/C	wv-ELISA^A^	0 (0/10)^1^	30 (3/10)^b^	100 (10/10)*	100 (10/10)*	100 (10/10)	100 (10/10)*	100 (10/10)	100 (10/10)*
	IFA^B^	0 (0/10)	10 (1/10)^b^	70 (7/10)*	60 (6/10)*	100 (10/10)	50 (5/10)*	60 (6/10)	100 (10/10)*
	SVN^C^	0 (0/10)	100 (10/10)^a^	100 (10/10)*	100 (10/10)*	100 (10/10)	90 (9/10)*	90 (9/10)	70 (7/10)*
NV/C	wv-ELISA^A^	0 (0/10)	0 (0/10)	0 (0/10)	0 (0/10)	0 (0/10)	10 (1/10)	0 (0/10)	0 (0/10)
	IFA^B^	0 (0/10)	0 (0/10)	0 (0/10)	0 (0/10)	0 (0/10)	0 (0/10)	0 (0/10)	0 (0/10)
	SVN^C^	0 (0/10)	0 (0/10)	0 (0/10)	0 (0/10)	0 (0/10)	0 (0/10)	0 (0/10)	0 (0/10)
NV/NC	wv-ELISA^A^	0 (0/10)	20 (2/10)	0 (0/10)	0 (0/10)	0 (0/10)	0 (0/10)	0 (0/10)	0 (0/10)
	IFA^B^	0 (0/10)	0 (0/10)	0 (0/10)	0 (0/10)	0 (0/10)	0 (0/10)	0 (0/10)	0 (0/10)
	SVN^C^	0 (0/10)	0 (0/10)	0 (0/10)	0 (0/10)	0 (0/10)	0 (0/10)	0 (0/10)	0 (0/10)
RP/C	wv-ELISA^A^	0 (0/10)	0 (0/10)*	20 (2/10)^ab^	20 (2/10)*	100 (10/10)	100 (10/10)	100 (10/10)	100 (10/10)
	IFA^B^	0 (0/10)	0 (0/10)*	0 (0/10)^b^	10 (1/10)*	100 (10/10)	100 (10/10)	100 (10/10)	100 (10/10)
	SVN^C^	0 (0/10)	50 (5/10)*	80 (8/10)^a^	50 (5/10)*	100 (10/10)	100 (9/9)	100 (10/10)	100 (10/10)
RPAdj/C	wv-ELISA^A^	0 (0/10)	0 (0/10)*	70 (7/10)*	60 (6/10)*	100 (10/10)	100 (10/10)	100 (10/10)	100 (10/10)
	IFA^B^	0 (0/10)	0 (0/10)*	50 (5/10)*	100 (10/10)*	100 (10/10)	100 (10/10)	100 (10/10)	100 (10/10)
	SVN^C^	0 (0/10)	60 (6/10)*	100 (10/10)*	100 (10/10)*	100 (10/10)	100 (10/10)	100 (10/10)	100 (10/10)

^A^Samples with a sample/positive (S/P) ratio greater than 0.14 were considered positive. Samples with a S/P ratio less than 0.14 were considered negative

^B^Samples with an IFA reciprocal titer greater than or equal to 40 were considered positive. Samples with an IFA titer less than 40 were considered negative

^C^Samples with a VN titer greater than or equal to 20 were considered positive. Samples with a VN titer less than 20 were considered negative

^1^Antibody positive/treatment group size; *wv-ELISA* Whole virus ELISA, *IFA* Indirect fluorescence antibody, *SVN* Serum virus neutralization

## Discussion

Porcine parainfluenza virus type-1 was originally detected in rectal and nasopharyngeal swabs by RT-rtPCR from slaughter swine in Hong Kong in 2013 [2]. PPIV-1 has since been detected worldwide from commercial swine in North and South America, Europe, and other parts of Asia [2, 3, 6, 7]. Although PPIV-1 appears widespread, serology assays for the detection of anti-PPIV-1 antibodies in serum and oral fluids were not available. In addition, little is known about the genetic and antigenic diversity of PPIV-1 or potential antibody cross-reactivity between different strains of virus. Preliminary data from the Iowa State University Veterinary Diagnostic Laboratory (ISU VDL, unpublished data) and prior reports [2, 4] suggest there is some genetic diversity between different strains of PPIV-1 worldwide. Although no gold standard PPIV-1 antibody assay currently exists for swine, the use of samples of known PPIV-1 infection status, antibody curves and ROC analysis provided the data to establish validated antibody assays and their corresponding cut-off values.

The IFA is a serology test used in many diagnostic laboratories to detect host-derived antibody due to its ease of development and high level of specificity. The three most common IFA assays used in domestic swine are directed against porcine reproductive and respiratory syndrome virus (PRRSV), porcine epidemic diarrhea virus (PEDV), and porcine circovirus-2 (PCV2) [9–11]. While the anti-PPIV-1 IFA developed in this study had equal specificity as the wv-ELISA, our results showed a significant decrease in sensitivity of the IFA relative to the wv-ELISA. This may be due to inherent differences in efficiency of viral antigen presentation between the two assays and/or detection methods or assay optimization. While both assays in this study are based on the MN16 strain and target anti-IgG antibodies, each uses different sources of secondary antibodies for fluorescence (IFA) or colorimetric (ELISA) visualization.

Serum virus neutralization assays are considered the reference method for detecting functional antibodies capable of preventing viral adsorption. Several different types of neutralization assays exist including the plaque reduction neutralization assay (PRN) [12], fluorescent focused neutralization assay (FFN) [13], and SVN assay [14]. Another important, functional assay that can be used for some viruses is the hemagglutination inhibition (HI) assay. Hemagglutination inhibition assays are designed to measure the decrease in hemadsorption based on antibody titers. In cattle, parainfluenza HI assays are typically performed due to the substantial decrease in turnaround time compared to conventional SVN [15]. However, PPIV-1 is unable to agglutinate rooster, turkey, or guinea pig red blood cells (unpublished data) precluding the use of HI assays for PPIV-1 antibody in swine. Virus neutralization assays have many potential applications including serving as a correlate of vaccine efficacy, identification of recent exposure with acute and convalescent sera, and serotyping if appropriate monoclonal antibodies are available. Neutralization assays are highly strain-specific and may not be ideal for general diagnostic use when there is strain-diversity in the field, but rather more appropriately used for farm-level applications [16]. Previously described plaque neutralization tests for antibody against HPIV-1 and HPIV-3 in marmosets showed seroconversion occurring around 10–14 days after infection [12]. In addition, HI and SVN antibodies have been detected against caprine parainfluenza virus type 3 (CPIV-3) at 7 and 14 DPI, respectively [17]. Whereas systemic bovine parainfluenza virus-3 (BPIV-3) neutralizing antibody in cattle could be detected as early as 6 days post inoculation [18]. These findings from other species are consistent with the PPIV-1 SVN results reported here. Additionally, earlier antibody detection by SVN compared to wv-ELISA or IFA could be isotype specific or due to virus specific factors. Systemic neutralizing antibodies can be IgM and IgG that may contribute to detection of an early immune response in contrast to the IFA and wv-ELISA tests that specifically detect IgG antibodies. Regarding other porcine viruses, prior reports have shown that SVN is considered less sensitive for PRRSV due to the slow onset of neutralizing antibodies, whereas SVN detects antibody earlier for PEDV compared to IFA or ELISA [19, 20].

The wv-ELISA, also referred to as enzyme immunoassay (EIA), have increased in popularity due to their ease of use, ability to perform as high throughput assays and their high level of sensitivity. Single-dilution wv-ELISA used to detect parainfluenza antibodies have been developed for a variety of species including cattle [21], monkeys [22], dogs [23], goats and sheep [24, 25] and humans [26]. Our results showed that the wv-ELISA had a similar TPR and TNR to the SVN assay and was significantly increased from the TPR and TNR demonstrated by the IFA. However, the PPIV-1 wv-ELISA did not detect seroconversion as early as SVN. The wv-ELISA, like neutralization assays, are commonly used to monitor for seroconversion post-vaccination or confirm recent infection. However, wv-ELISA is more suited for serosurveillance and general diagnostic applications compared to neutralization assays [16]. The diagnosis of parainfluenza virus infection based on antibody detection in paired sera collected two weeks apart is complicated by endemic infections, maternal antibody, and routine vaccination under field conditions [27]. The use of whole virus antigen for indirect wv-ELISA has been shown to generally be more sensitive [28, 29] but less specific [30] than recombinant antigen due to the potential for binding to non-target, cellular and viral proteins [31].

## Conclusions

Three PPIV-1 antibody assays, IFA, wv-ELISA and SVN, performed similarly under experimental conditions with serum samples of known status. The SVN detected seroconversion earlier compared to either the IFA or the wv-ELISA. Both the wv-ELISA and the SVN had similar diagnostic performance, while the IFA demonstrated less sensitivity compared to the wv-ELISA. All three assays are considered to perform well based on the conditions of this study and can be implemented in routine diagnostic use depending on the application. These assays will be important for future studies to determine seroprevalence and vaccine efficacy against PPIV-1 under field conditions. Ongoing surveillance studies will be required to monitor the need for strain updates in the respective assays.

## Materials and methods

### Viruses and cells

The USA/MN25890NS/2016 (MN16) and USA/IA/84915LG/2017 (IA17) PPIV-1 were propagated in either MK2 cells (Studies A and B) or swine testicular (ST) cells (Study C) at a multiplicity of infection (MOI) of 0.01 in post inoculation medium (PIM) consisting of minimum essential medium (MEM; Gibco™, Waltham MA) supplemented with 1 μg/mL of L-(tosylamido-2-phenyl) ethyl chloromethyl ketone treated trypsin (TPCK; Worthington Biochemical, Lakewood NJ), 1% penicillin–streptomycin, 1% L-glutamine, 0.1% Amphotericin B, and 0.1% gentamicin (Gibco™, Waltham MA). The cells were washed with phosphate buffered saline twice (PBS; Gibco™ Waltham, MA), and the virus was adsorbed on the cells for 2 h. The inoculum was diluted to a titer of approximately 10^5^ TCID_50_/mL in PIM prior to inoculation. Titers have been shown to be roughly equivalent between the two cell lines (unpublished data). Virus titers were calculated using the Reed Muench method [32]. Each inoculum was titrated by tenfold serial dilutions in quintuplicate using the following concentrations: 10^–1^, 10^–2^, 10^–3^, 10^–4^, 10^–5^, 10^–6^, 10^–7^, 10^–8^. MK2 cells (ATCC® CCL-7™) were grown in 1% equine serum (Gibco™, Waltham MA) and 1% penicillin–streptomycin. ST cells (ATCC® CRL-1746) were grown in MEM (Thermo-Fisher Scientific, Waltham MA) supplemented with 10% Fetal Bovine Serum (FBS; Atlas Biologicals, Fort Collins CO), 1% penicillin–streptomycin, 1% L-glutamine, 0.1% Amphotericin B, and 0.1% gentamicin (Gibco™, Waltham MA).

### Experimental designs

A total of 576 serum samples were obtained from experimentally inoculated or vaccinated piglets approximately 3–6 weeks of age. However, only 521 samples were tested by all three assays. The serum of known exposure status was obtained from three separate, longitudinal challenge studies, denoted A-C. Specific details regarding the experimental design for each study are outlined in the subsequent sections. All studies were conducted over the span of two years from 2017 to 2018. Blood was collected in BD Vacutainer® SST™ blood collection tubes (BD, Franklin Lakes, NJ) by venipuncture of the anterior vena cava as it exits cranial to the thoracic inlet. The blood was allowed to clot for at least 20 min before centrifuging at 3,000 × *g* for 10 min in serum separator tubes and poured off in 5 mL snap cap tubes (Fisher Scientific, Waltham MA) for storage at -80 °C.

### Study A: PPIV-1 Pathogenesis Study in Cesarean-Derived Colostrum-Deprived (CDCD) Piglets

Fourteen CDCD pigs at 6–7 weeks of age were randomly assigned into challenge (*N* = 10) and non-challenge (*N* = 4) groups and confirmed negative for circulating PPIV-1 antibody using a non-validated ELISA and shedding by RT-rtPCR in nasal swabs (NS) prior to challenge. Piglets in the challenge group (Ch) were inoculated 2 mL intratracheally and 2 mL intranasally with tissue culture isolate MN16 at a concentration of 6.3 × 10^4^ TCID_50_/mL; similarly, piglets in the non-challenge (NCh) group received MEM (Gibco™, Waltham, MA) sham-control at the same volume and route of inoculation. Necropsies occurred at 2, 5, and 27 DPI with 3 Ch and 1 NCh pigs necropsied at 2 DPI, 4 Ch and 1 NCh pigs necropsied at 5 DPI, and 3 Ch and 2 NCh pigs necropsied at 27 DPI. In total, 69 serum samples (45 Ch, 24 NCh) were collected from available pigs at 0, 3, 5, 7, 9, 13, 16, 21, 24, 27 DPI. However, 34 samples (22 Ch, 12 NCh) were tested by all three assays (Table 5).

### Study B: HPIV-1 and PPIV-1 isolate comparison in conventional piglets

Thirty, conventional, 4-week-old pigs were randomly selected from a high-health farm confirmed negative from active PPIV-1 infection by RT-rtPCR [4] and negative for maternal antibodies using a non-validated wv-ELISA. Pigs were blocked by weight and randomly assigned into three Ch groups of 8 pigs each consisting of human parainfluenza virus type 1 (HPIV-1) (ATCC® VR-94™), PPIV-1 USA/MN25890NS/2016 (MN16) or USA/IA/84915LG/2017 (IA17) and a NCh group of 6 pigs. The challenge material was administered as described in Study A, with 2 mL administered by the intratracheal route and 2 mL by the intranasal route at a titer of approximately 10^5^ TCID_50_/mL. The 6 pigs in the NCh group were given a sham MEM control (Gibco™, Waltham MA). At 5 DPI, 5 pigs were necropsied in the HPIV-1, MN16, and IA17 groups and 4 pigs were necropsied from the NCh group to evaluate pathogenesis while 3 pigs from each of the Ch groups and 2 from the NCh control group were necropsied at 28 DPI to allow time to evaluate seroconversion (9 Ch, 2 NCh). A total of 107 serum samples were collected from the study at 0, 7, 10, 14, 17, 21, 24, 28 DPI. However, 88 samples (72 Ch, 16 NCh) were tested by the three assays (Table 6).

### Study C: Vaccination of conventional piglets with an RNA particle vaccine

Fifty, conventional, 4-week-old pigs were randomly selected from a high-health farm confirmed negative from active PPIV-1 infection and maternal antibodies in a similar manner as described in studies A and B. Pigs were randomly allocated into 5 groups of 10 pigs denoted nonvaccinated/nonchallenged (NV/NC), nonvaccinated/challenged (NV/C), RP vaccinated/challenged (RP/C), adjuvanted RP vaccinated/challenged (RPAdj/C), and live exposure/challenged (LE/C) groups. The replicon particle vaccine platform has been extensively characterized for many viruses including swine influenza A virus (IAV) [33, 34]. Piglets were vaccinated or exposed to PPIV-1 at 0 and 24 DPV. Serum samples were collected on 0, 11, 18, 24, 31, 38, 43, and 48 DPV from each pig. On 43 DPV, piglets were challenged with approximately 10^4.8^ TCID_50_/mL of PPIV-1 isolate IA17 with 2 mL of virus intranasally and 2 mL intratracheally. All piglets were euthanized at 5 DPI. Fifty samples were collected per timepoint (20 vaccinated, 10 live exposure, 20 non-vaccinated) for a total of 400 serum samples. However, 399 samples (19 vaccinated, 10 live exposure, 20 non-vaccinated) were tested by all three assays (Table 7).

### SVN assay development

Serum samples were aliquoted into deep, 96-well plates and inactivated at 56 °C for 30 min. MK2 cells were seeded on 96-well plates and grown to 95% confluency in M199 medium (Gibco™, Waltham, MA) supplemented with 1% equine serum (MilliporeSigma, Burlington, MA) and 1% pen-strep (Gibco™, Waltham, MA). A separate set of 96-well plates used for dilution were prepared by adding PIM containing 1 µg/mL TPCK (Worthington Chemical Corporation, Lakewood, NJ) trypsin and 1% pen-strep.

Sera were serially diluted in PIM in the following dilution series: 1:10, 1:20, 1:40, 1:80, 1:160, 1:320: 1:640, 1:1280. Selected virus (MN16 or IA17 corresponding to challenge inoculum) at a known titer was diluted to 200 TCID_50_/well, equivalent to 4 × 10^3^ TCID_50_/mL. PPIV-1 virus control (infection control) and PIM non-virus control (media only control) columns were included on each plate. An equal volume of stock virus was added to all columns except the non-virus control, and the plate was incubated at 37 °C for 1 h. As the serum-virus mixture was incubating, the cells were washed twice with pre-warmed MEM (Gibco™, Waltham, MA) without antibiotics or trypsin. The remaining media on the cell monolayer was decanted and 100 µL of the serum-virus mixture was transferred to the cells. The serum-virus mixture was then allowed to incubate on the cells at 37 °C for 2 h. The mixture was removed and the cells were washed twice with 125 µL base medium. The last wash of 100 µL PIM remained on the cells to incubate for 72 h, after which the cells were fixed with 80% acetone (MilliporeSigma, Burlington, MA) at -20ºC for 15 min. The cells were stained with a horseradish peroxidase protocol as described previously [4]. The titer was determined as the reciprocal of the highest serum dilution resulting in ≥ 95% reduction in infectivity. Serum samples with SVN titer ≥ 20 were considered positive and < 20 negative.

### IFA assay development

An IFA test was developed by modifying existing protocols from peer reviewed literature and ISU VDL protocols [13, 35]. ST cells (ATCC® CRL-1746™) were seeded on 96-well microplates at a concentration of approximately 1.5–2 × 10^4^ cells per well and incubated at 37ºC with 5% CO_2_. The ST cells were grown in MEM (Gibco™, Waltham, MA) supplemented with 10% fetal bovine serum (FBS; Atlas Biologicals, Fort Collins MO), 1% Pen-strep, L-glutamine (Gibco™, Waltham, MA), and amphotericin B (Gibco™, Waltham, MA), until they reached 98% confluency. After reaching confluency, the cells were washed three times with PIM containing 1 µg/mL TPCK trypsin and inoculated with PPIV-1 isolate MN16 at various MOIs and incubated for various times to optimize conditions. Specifically, MOI of 0.005, 0.01, 0.025, 0.05, 0.1 and incubation times of 6, 12, 18, 24, 36, and 48 h were evaluated with a checkerboard scheme. The plates were fixed with 80% cold acetone, incubated with 50 µL of primary mouse monoclonal antibody and stained with 50 µL goat anti-mouse secondary antibody conjugated to fluorescein isothiocyanate (FITC; Southern Biotech, Birmingham, AL) to evaluate cell infection status. The assay conditions were chosen when approximately 50% of cells were infected and the foci could be easily distinguished from background fluorescence. Ultimately, the optimized conditions to prepare PPIV-1 infected IFA plates utilized a MOI of 0.05 for PPIV-1 inoculation and 24 h for incubation at 37 °C. Following this optimized protocol, PPIV-1 IFA plates were prepared and stored at -20ºC for up to one week prior to testing experimental serum samples.

The prepared IFA plates were brought to room temperature and incubated at 37 ºC for 1 h in phosphate buffered saline (PBS) containing 0.05% Tween 20 (PBST) and 1% bovine serum albumin (BSA) (Jackson ImmunoResearch Inc., West Grove, PA USA) to reduce nonspecific binding. A final volume of 50 µL of two-fold serial dilutions of serum in PBST and BSA (1:20, 1:40, 1:80, 1:160, 1:320, 1:640, 1:1,280, 1:2,560, 1:5,120, 1:10,240, 1:20,480, and 1:40,960) were loaded into 96-well microplates. The test sera were then incubated for 1 h at 37ºC before being washed four times with PBST with 5 min soaks between each wash.

The plates were incubated with a FITC conjugated goat anti-porcine IgG (Southern Biotech, Birmingham, AL) diluted 1:200 in PBST and BSA at 37ºC for 1 h followed by four washes with PBST and BSA. The plates were dried in the dark at room temperature and evaluated using an Olympus IX71 fluorescence microscope (Olympus America Inc., Center Valley, PA USA) at a wavelength of 494 nm in a dark room. The endpoint dilution was determined when cell-specific staining was not discernable against background fluorescence. Samples with an IFA antibody titer ≥ 40 were considered positive and < 40 as negative.

### wv-ELISA assay development

A PPIV-1 isolate (MN16) was amplified in ST cells, each batch containing approximately 500 mL of virus, as described earlier. Propagated PPIV-1 was subjected to one freeze–thaw cycle (-80 °C), and the harvested material were centrifuged at 4,000 × g for 15 min to remove cell debris. The clarified supernatant was ultracentrifuged at 140,992 × *g* for 3 h and washed twice with phosphate buffered saline (1X PBS, Gibco®, Thermo Fisher Scientific) pH 7.4 to remove cell culture media components. The pellet was then resuspended in 100 µL PBS at 1:100 dilution from the original media volume and stored at -80ºC. The optimum dilution was determined using a checkerboard titration based on known antibody positive and negative sera to maximize signal while minimizing background noise. For coating, polystyrene 96-well ELISA plates (Nunc, Maxisorp, Thermo Fisher Scientific, Agawam, MA) were coated with 100 µL of the whole virus solution at optimum dilution (1:200 in PBS) per well and incubated at 4ºC for 16 h. The plates were washed five times with 300 µL per well of PBST (0.1% Tween 20 in PBS), blocked with 300 µL per well of a blocking solution containing 1% BSA (w/v) (Jackson ImmunoResearch, West Grove, PA USA), and incubated at room temperature (RT, 20-25ºC) for 2 h. Next, the blocking solution was removed with no wash step, and the plates were dried at 37ºC for 4 h before packing in sealed bags with desiccant packs and stored at 4ºC.

Serum samples were tested at 1:100 dilution (100 µL reaction volume) in a sample diluent containing 40% of newborn calf serum (Gibco®, Waltham, MA), incubated at RT (20-25ºC) for 1 h, and washed 5 times with PBS-T. Then, 100 μL of horse-radish peroxidase (HRP)-conjugated goat anti-pig IgG (Fc) antibody (Bethyl Laboratories Inc., Montgomery, TX) diluted 1:20,000 were added to each well and the plates were incubated at RT for 1 h. After a washing step, the reaction was visualized by adding 100 μL of tetramethylbenzidine-hydrogen peroxide (TMB, Surmodics IVD, Inc., Eden Prairie, MN, USA) substrate solution to each well, and incubated for 10 min at RT. The reaction was stopped by adding 100 μL of stop solution (Surmodics IVD, Inc.) to each well, and the optical density was read at 450 nm using an ELISA reader (Molecular Devices, Sunnyvale, CA). ELISA antibody responses were expressed as sample-to-positive (S/P) ratios.

$$SP Ratio:\frac{\left(sample OD-negative control mean OD\right)}{(positive control mean OD-negative control mean OD)}$$

### Statistical analysis

Statistics were performed with the open-source statistical software R version 4.0.3. Differences in proportions were analyzed by the Cochrane Q test available in the RVAideMemoire package (v0.9–79). If significant, pairwise comparisons were conducted by using individual McNemar tests of association available in the rcompanion package (v 2.4.1) with a Bonferroni adjustment for multiple comparisons. Receiver operating characteristic (ROC) curves were constructed, analyzed, and graphed with the pROC package (v1.16.2) [8]. Area under the curve were estimated for each assay using the empirical ROC curve [8]. Correlation between assay results and computation of R^2^ were conducted using the lm function in base R. Results were visualized with the ggplot2 library version 3.3.2. Difference in AUC between assay results and confidence intervals for sensitivities and specificities for selected cutoff values were evaluated using bootstrap methods which considers the repeated measures data structure.

## Data Availability

The data that support the findings of this study are available upon request for Studies A and B. Restrictions apply to the availability for Study C data, which were used under license for the current study, and are not publicly available. However, data are available from the authors upon reasonable request and with permission of Merck & Co., Inc., Kenilworth, NJ, USA for Study C.

## Abbreviations

AUC: Area under the curve
BPIV-3: Bovine parainfluenza virus-3
BSA: Bovine serum albumen
CDCD: Cesarean derived colostrum deprived
Ch: Challenged
DPI: Days post inoculation
DPV: Days post vaccination
EIA: Enzyme immunoassay
FBS: Fetal bovine serum
FFN: Fluorescent focused neutralization assay
FITC: Fluorescein isothiocyanate
HI: Hemagglutination inhibition assay
HPIV-1: Human parainfluenza virus 1
IA17: USA/IA/84915LG/2017
IFA: Indirect fluorescence antibody
ISU VDL: Iowa State University Veterinary Diagnostic Laboratory
MEM: Minimum essential medium
MK-2: Rhesus monkey kidney cells
MN16: USA/MN25890NS/2016
MOI: Multiplicity of infection
NCh: Non-challenged
NS: Nasal swabs
NV/C: Nonvaccinated/challenged
NV/NC: Nonvaccinated/nonchallenged
PBS: Phosphate buffered saline
PBST: Phosphate buffered saline with Tween 20
PCV2: Porcine circovirus 2
PEDV: Porcine epidemic diarrhea virus
PIM: Post inoculation medium
PPIV-1: Porcine parainfluenza virus 1
PRN: Plaque reduction neutralization assay
PRRSV: Porcine reproductive and respiratory syndrome virus
ROC: Receiver operating characteristic curve
RP: RNA Particle
RP/C: RP vaccinated/challenged
RPAdj/C: Adjuvanted RP vaccinated/challenged
RT: Room temperature
RT-rtPCR: Reverse transcription real time polymerase chain reaction
ST: Swine testicular
SVN: Serum virus neutralization
TCID50/ml: 50% Tissue culture infectious dose per milliliter
TNR: True negative rate
TPCK: L-(tosylamido-2-phenyl) ethyl chloromethyl ketone
TPR: True positive rate
US: United States
wv-ELISA: Whole virus enzyme linked immunosorbent assay
